# Unraveling the Role of Maternal Serum Ferritin Levels in Preterm Delivery: A Comprehensive Review

**DOI:** 10.7759/cureus.54515

**Published:** 2024-02-20

**Authors:** Anubha Dande, Sandhya Pajai, Aishwarya Gupta, Seema Dande, Neha Sethi

**Affiliations:** 1 Obstetrics and Genecology, Jawaharlal Nehru Medical College, Datta Meghe Institute of Higher Education & Research, Wardha, IND; 2 Obstetrics and Gynecology, Jawaharlal Nehru Medical College, Datta Meghe Institute of Higher Education & Research, Wardha, IND

**Keywords:** public health, risk factors, pregnancy, iron status, maternal ferritin, preterm delivery

## Abstract

Preterm delivery remains a critical global health concern, with numerous adverse consequences for both neonate and healthcare systems. Understanding the relationship between maternal ferritin levels, as a marker of iron status, and the risk of preterm birth is the focal point of this comprehensive review. We provide insights into the multifaceted nature of this connection, highlighting factors that influence maternal ferritin levels, including dietary intake, genetic and physiological variations, comorbidities, and iron supplementation. While evidence suggests an association between low maternal ferritin levels and preterm birth, causality remains elusive, necessitating further research with robust study designs. The potential mechanisms linking maternal iron status to preterm birth, such as inflammation, infection, and oxidative stress, are explored, underscoring the need for in-depth investigations. This comprehensive review emphasizes the clinical importance of assessing and monitoring maternal ferritin levels in prenatal care and advocates for public health initiatives to raise awareness and provide targeted interventions, particularly in high-risk populations. As we strive to address these unanswered questions and embark on innovative research directions, the aim is to ultimately enhance our understanding of the complex relationship between maternal iron status and preterm birth, leading to improved maternal and child health outcomes.

## Introduction and background

Preterm delivery, defined as giving birth before 37 weeks of gestation, remains a significant global public health concern. It is a leading cause of neonatal morbidity and mortality, contributing to long-term health issues for premature infants. According to the World Health Organization (WHO), an estimated 15 million babies are born prematurely each year, with preterm birth complications being responsible for approximately one million infant deaths annually. The occurrence of preterm delivery has both immediate and long-lasting implications for the affected neonate and their families [1]. Preterm birth can lead to a range of health challenges, including respiratory distress syndrome, infections, developmental delays, and increased risks of chronic diseases later in life. These adverse outcomes place emotional and financial burdens on families and strain healthcare systems and resources. Understanding the risk factors and mechanisms behind preterm delivery is crucial for developing effective preventative measures and improving maternal and child health outcomes [2].

During pregnancy, the mother's nutritional status plays a pivotal role in ensuring the healthy development and well-being of the fetus. Adequate nutrient intake is necessary for the growth and development of the placenta and the developing fetus. Insufficient nutrition can result in intrauterine growth restriction and an increased risk of preterm birth. Iron, an essential mineral, is of particular importance during pregnancy as it is vital for oxygen transport, energy production, and overall fetal development [3]. Ferritin, a protein complex found in cells throughout the body, serves as a critical indicator of an individual's iron status. Within pregnancy, maternal ferritin levels indicate the body's iron stores and can provide insights into potential iron deficiency or excess. While ferritin is not the only measure of iron status, it is one of the most commonly used markers due to its sensitivity to changes in iron stores [4].

Low maternal ferritin levels can indicate an iron deficiency, which, if left unaddressed, can lead to anemia and other health complications during pregnancy. Conversely, elevated ferritin levels may suggest iron overload or inflammation, adversely affecting maternal and fetal health. Understanding the relationship between maternal ferritin levels and preterm delivery is a critical area of research that can shed light on the importance of iron status during pregnancy [5]. This comprehensive review aims to examine the existing body of literature on the role of maternal serum ferritin levels in preterm delivery. By synthesizing and analyzing the available evidence, this review aims to provide a deeper understanding of the relationship between maternal iron status, as reflected by ferritin levels, and the risk of preterm birth. We will explore the potential mechanisms that connect iron status to preterm delivery and discuss the various factors that can influence maternal ferritin levels during pregnancy. Additionally, we will address the clinical implications of these findings and highlight areas that require further research.

## Review

Iron metabolism and ferritin

Overview of Iron Metabolism in the Human Body

Dietary iron intake: Iron is an essential mineral crucial for various physiological processes within the human body. It is primarily obtained through dietary sources, available in heme and non-heme iron. Heme iron is primarily found in animal-based foods such as red meat, poultry, and fish. It is notable for its higher bioavailability, meaning the body more efficiently absorbs it. In contrast, non-heme iron is present in plant-based foods like legumes, grains, and leafy greens. The body less readily absorbs this iron, and various dietary components and factors can influence its absorption [6].

Absorption: The small intestine plays a central role in iron absorption. This is where dietary iron enters the body, whether heme or non-heme. In the small intestine, iron is absorbed primarily in its ferrous (Fe2+) form. Iron absorption is highly regulated, ensuring the body takes enough iron to meet physiological demands [7].

Transportation: Once absorbed, iron is transported in the bloodstream to a protein called transferrin. Transferrin acts as a carrier, ferrying iron to various tissues and organs throughout the body. This ensures that iron is distributed to where it is needed, especially for processes like hemoglobin synthesis in red blood cells, which is crucial for oxygen transport [8].

Storage: Iron is not used by the body immediately upon absorption; therefore, excess iron is stored as a reserve for future needs. The primary sites of iron storage are the liver, spleen, and bone marrow. This stored iron is held in a form known as ferritin, which acts as a reservoir that can be tapped into when the body's iron demands increase, such as during periods of rapid growth or increased blood production [9].

Recycling: Iron is a finite resource, and the body has an efficient recycling system to ensure that iron is used effectively. Macrophages, specialized immune cells, break down senescent or aged red blood cells. These macrophages recover the iron from the hemoglobin of the degraded red blood cells and return it to circulation for reuse. This recycling process minimizes the need for continuous dietary iron intake and helps maintain iron balance in the body [10].

Role of Ferritin as an Iron Storage Protein

Ferritin structure: Ferritin, as a fundamental component of iron storage in the human body, is a remarkable protein with a highly organized structure. It comprises two subunits, namely H (heavy) and L (light), which combine to create a spherical cage-like structure. This hollow sphere serves as a safe and efficient repository for iron ions. The structure of ferritin prevents iron ions from precipitating as insoluble deposits, ensuring that they remain in a soluble and non-toxic form within the protein's core [11].

Iron binding: One of the most crucial functions of ferritin is its capacity to bind and store iron. This protein has the remarkable ability to store thousands of iron atoms within its spherical structure. Importantly, ferritin binds iron primarily in the form of ferric ions (Fe3+), converting them into a less reactive and non-toxic form. This binding allows for the efficient storage of excess iron. It prevents the potentially harmful effects of free iron ions, which can trigger oxidative damage and other detrimental reactions in the body [11].

Regulation: The body meticulously regulates Ferritin synthesis and degradation in response to its iron requirements. When the body's iron stores are low, a feedback mechanism triggers the upregulation of ferritin synthesis. This means more ferritin is produced, and more iron is captured and stored within ferritin molecules. Conversely, ferritin production is reduced when iron stores are deemed sufficient or in excess to prevent further iron storage. This dynamic regulation helps maintain iron balance and ensures that iron resources are available when needed, such as during periods of increased demand, like pregnancy [12].

Diagnostic value: Serum ferritin levels are commonly measured in clinical settings to assess an individual's iron status. The typical serum ferritin levels can vary across populations and laboratories but generally fall within these ranges: For adult males, 12 to 300 nanograms per milliliter (ng/mL), and for adult females, 12 to 150 ng/mL. Another set of ranges includes 24 to 336 ng/mL for adult males and 24 to 307 ng/mL for adult females. Additionally, adult females may have levels ranging from 11 to 307 micrograms per liter [13]. The diagnostic value of ferritin lies in its ability to indicate iron stores within the body. Elevated serum ferritin levels can indicate conditions such as iron overload or inflammation. On the other hand, low serum ferritin levels are typically suggestive of iron deficiency, which can lead to anemia and other health complications. These diagnostic insights help healthcare providers make informed decisions regarding iron status and guide interventions like dietary modifications or iron supplementation to address iron-related issues in patients [13].

Regulation of Ferritin Levels in Pregnancy

Hormonal changes: Pregnancy initiates complex hormonal changes essential for supporting the developing fetus and maintaining maternal health. One significant hormonal alteration is the increase in hepcidin levels. Hepcidin is a crucial regulator of iron homeostasis in the body. During pregnancy, the maternal body produces higher hepcidin levels, potentially influencing iron metabolism. Hepcidin exerts its effects by limiting the absorption of dietary iron from the small intestine and reducing the release of iron from storage sites such as the liver and spleen. While this mechanism protects the mother from iron deficiency, it can sometimes lead to suboptimal iron availability for the developing fetus [14].

Ferritin synthesis: In response to the increased iron demands during pregnancy, maternal ferritin synthesis may be upregulated. Ferritin is the primary protein responsible for storing iron within the body. When iron stores are deemed insufficient, the body increases ferritin production to capture and store more iron. This response serves as a protective mechanism to ensure an adequate supply of iron for the growing fetus and to meet the increased maternal blood volume and other physiological changes. Consequently, elevated ferritin levels are often observed in pregnant women, reflecting the heightened requirement for iron storage [15].

Diagnostic considerations: The dynamic hormonal changes and increased ferritin synthesis during pregnancy introduce specific challenges when interpreting maternal ferritin levels. Elevated ferritin levels in pregnant individuals may not necessarily indicate iron overload, as they can result from the body's adaptive response to the heightened iron demands of pregnancy. Conversely, low ferritin levels in pregnancy may not always reflect true iron deficiency. Maternal iron status is complex and multifactorial, influenced by dietary intake, physiological changes, and the intricate interplay of hormones. Thus, healthcare providers must consider these dynamic factors when assessing iron status during pregnancy and making clinical decisions about supplementation or dietary recommendations to support maternal and fetal health [16].

Maternal ferritin and preterm delivery

Epidemiology of Preterm Delivery

Global prevalence: Preterm birth is a global public health concern with significant variation in prevalence among countries and regions. The World Health Organization (WHO) estimates that approximately 10% of all births worldwide are preterm. However, this prevalence can vary widely, with some regions experiencing higher rates than the global average. Understanding these variations is essential for targeting interventions and resources to areas with the greatest need and reducing the overall burden of preterm birth on maternal and child health [17].

Risk factors: Preterm delivery is a multifactorial phenomenon, and various risk factors contribute to its occurrence. These factors include maternal age, with both very young and older mothers at higher risk, as well as multiple pregnancies (e.g., twins or triplets), which are associated with increased prematurity rates. Infections during pregnancy, maternal medical conditions such as diabetes and hypertension, and lifestyle factors like smoking and substance abuse can also elevate the risk of preterm birth. Recognizing and addressing these risk factors is crucial for preventive strategies and early interventions to reduce preterm birth rates [18].

Health consequences: Preterm birth can have profound health consequences for the newborn, including the increased risk of conditions such as respiratory distress syndrome, developmental delays, and long-term health issues. These health consequences not only affect the quality of life for the child but also place a substantial burden on healthcare systems and families. Efforts to prevent preterm birth, improve neonatal care, and provide ongoing support and interventions for preterm infants are critical to mitigate the health and socioeconomic impacts associated with preterm delivery. By addressing these consequences, we can work toward better maternal and child health outcomes and a more sustainable healthcare system [19].

Previous Studies on Maternal Ferritin and Preterm Delivery

Observational studies: The investigation into the association between low maternal ferritin levels and preterm delivery has primarily relied on observational studies. These studies have sought to uncover whether a link exists between maternal iron status, as reflected by ferritin levels, and the risk of preterm birth. However, the results of these studies have often been mixed, reflecting the intricate nature of this relationship. The challenges inherent in observational designs, such as the potential for selection bias and the complexity of isolating specific causal pathways, have contributed to the diversity of findings [20].

Meta-analyses: In an attempt to provide a more comprehensive understanding of the relationship, several meta-analyses have been conducted. These analyses aim to synthesize data from multiple observational studies, pooling their findings to derive more robust conclusions. However, even meta-analyses have yielded varying results, with some suggesting a significant association between low maternal ferritin levels and preterm delivery. In contrast, others have found evidence inconclusive or insufficient to establish a firm connection. The heterogeneity of included studies, differences in study populations, and variations in methodology can all contribute to the disparities in meta-analytic outcomes [21].

Confounding factors: The association between maternal ferritin levels and preterm delivery is influenced by various confounding factors that complicate the interpretation of study results. These factors include maternal age, nutritional status, the presence of comorbidities (e.g., diabetes, hypertension), and geographic location. Researchers must carefully account for and control these confounding variables in their analyses to disentangle the true relationship between maternal ferritin levels and preterm birth. The failure to address these confounders adequately can lead to spurious or misleading associations [22].

Potential Mechanisms Linking Low Maternal Ferritin to Preterm Delivery

Hypoxia and placental function: Iron deficiency can have significant implications for maternal health and fetal development, primarily through its potential to induce hypoxia. Hypoxia, characterized by low oxygen levels in the blood, can result from iron deficiency. When a pregnant individual experiences hypoxia due to inadequate iron levels, it can impact placental function. The placenta is a critical organ during pregnancy, facilitating the exchange of oxygen and nutrients between the mother and the developing fetus. Insufficient oxygen and nutrient supply from the placenta can lead to fetal growth restriction and developmental delays, raising the risk of preterm birth. This emphasizes the importance of maintaining adequate iron levels to ensure the proper functioning of both maternal and fetal physiological processes [23].

Inflammation and infection: Iron deficiency can compromise the maternal immune system, rendering pregnant individuals more susceptible to infections and inflammation. Infections and inflammation are well-established risk factors for preterm birth. Iron deficiency can weaken the immune response, impair the body's ability to fight pathogens, and disrupt the balance of immune processes. This, in turn, can lead to infections that may directly affect the uterus and induce inflammation. Inflammatory responses can stimulate uterine contractions and promote preterm labor. Consequently, addressing iron deficiency is essential for maintaining maternal health and reducing the risk of complications that can lead to preterm birth [24].

Oxidative stress: Iron deficiency can increase oxidative stress within the body. Oxidative stress arises when an imbalance exists between producing harmful reactive oxygen species (ROS) and the body's antioxidant defenses. Excessive oxidative stress can cause damage to cells, tissues, and DNA. When it occurs during pregnancy, it can potentially harm the developing fetus, affecting average fetal growth and development. This oxidative damage may disrupt the regulatory mechanisms that govern uterine contractions, thereby increasing the likelihood of preterm birth. The prevention and management of iron deficiency during pregnancy become critical for maternal health and protecting fetal development from the adverse effects of oxidative stress [25].

Uterine contractions: Iron deficiency may impact the uterine muscle's ability to contract and relax efficiently. Inadequate uterine contractions or irregularities in muscle function can lead to preterm labor. Effective uterine contractions are essential for the onset of labor and the progression of pregnancy to term. Alterations in uterine contractility, influenced by factors like iron deficiency, can result in premature contractions or an inability to sustain normal uterine tone. These irregularities can contribute to preterm birth, underscoring the significance of addressing iron deficiency as part of a comprehensive approach to reducing the risk of preterm delivery [26].

Role of Inflammation in Preterm Birth and Its Connection to Iron Status

Inflammatory pathways: Inflammation is a complex and tightly regulated process within the body. In the context of pregnancy and preterm labor, inflammation can trigger a cascade of events that promote uterine contractions and lead to preterm birth. Inflammatory responses may include the release of pro-inflammatory cytokines, signaling molecules that promote inflammation, and immune responses. These cytokines can stimulate the production of prostaglandins, substances known to induce uterine contractions. Additionally, inflammatory processes can contribute to the breakdown of the extracellular matrix, which provides structural support to the amniotic membranes. When this support weakens, it can lead to preterm rupture of the membranes, a common precursor to preterm birth. Therefore, activating inflammatory pathways is critical in understanding the mechanisms underlying preterm labor and birth [27].

Iron regulation and inflammation: Iron is essential for various physiological processes and plays a role in modulating the immune response. The relationship between iron status and inflammation is intricate. Iron is crucial for the proper functioning of immune cells, and alterations in iron availability can influence the balance of pro-inflammatory and anti-inflammatory factors. Low iron levels, as seen in iron deficiency, can lead to dysregulation of the immune response, potentially promoting inflammation. Inflammation-related complications during pregnancy, such as infections or inflammatory conditions, are known risk factors for preterm birth. Thus, there is a direct link between iron status and the potential for inflammation-related preterm birth. Maintaining appropriate iron levels during pregnancy is a critical consideration in mitigating the risk of preterm labor associated with inflammation [28].

Factors influencing maternal ferritin levels

Dietary Factors Affecting Maternal Iron Intake

Dietary iron sources: The source of dietary iron is a critical determinant of iron intake during pregnancy. There are two primary forms of dietary iron: heme iron and non-heme iron. Heme iron is predominantly found in animal-based foods such as red meat, poultry, and fish. It is more readily absorbed by the body, making it an efficient source of iron. In contrast, non-heme iron is present in plant-based foods like leafy greens, legumes, and fortified cereals. Non-heme iron is absorbed less efficiently. Therefore, the type of iron-rich foods in a pregnant person's diet significantly affects the amount of iron they can absorb and utilize. Understanding these differences is crucial for tailoring dietary recommendations to optimize iron intake during pregnancy [29].

Iron absorption enhancers and inhibitors: Iron absorption is influenced not only by the source of dietary iron but also by various dietary components that can enhance or inhibit absorption. For instance, vitamin C in fruits and vegetables enhances iron absorption. Therefore, consuming vitamin C-rich foods alongside non-heme iron sources can improve iron absorption. Conversely, certain compounds like calcium and tannins in dairy products and some plant-based foods can inhibit iron absorption. To optimize iron intake, it's essential to understand these interactions and make dietary choices that promote efficient iron absorption. This knowledge can help mitigate the risk of low maternal ferritin levels due to poor dietary iron absorption [29].

Nutritional deficiencies: Inadequate overall nutrition can contribute to iron deficiency during pregnancy. Malnourished individuals may not consume enough iron-rich foods, exacerbating the risk of low maternal ferritin levels. In some cases, diets may lack diversity, leading to deficiencies in essential nutrients, including iron. Additionally, diets that are low in bioavailable iron sources, such as heme iron, and high in inhibitors of iron absorption can further increase the risk of iron deficiency. Proper nutrition, rich in various nutrient-dense foods, is fundamental for ensuring that pregnant individuals receive an adequate intake of iron and other essential nutrients. Addressing nutritional deficiencies is vital for preventing iron deficiency anemia and maintaining optimal maternal iron status during pregnancy [23].

Genetic and Physiological Factors Impacting Iron Absorption and Storage

Genetic polymorphisms: Genetic polymorphisms, or variations in specific genes related to iron metabolism, can significantly impact an individual's ability to absorb and store iron. One example is the HFE gene, associated with hereditary hemochromatosis, characterized by excessive iron absorption and potential iron overload. Other genetic variations can result in reduced iron absorption or impaired iron utilization. Polymorphisms that affect iron metabolism may increase the risk of iron deficiency in affected individuals, as their bodies may struggle to maintain sufficient iron stores despite a seemingly adequate dietary intake. Recognizing these genetic factors can help identify those at higher risk of iron deficiency and tailor interventions accordingly [30].

Menstrual blood loss: In women, heavy menstrual bleeding can significantly contribute to the development of iron deficiency. During menstruation, women lose blood and iron with each cycle. Those with heavy or prolonged periods are at an increased risk of experiencing substantial iron loss, which may exceed their ability to replenish their iron stores through dietary sources alone. The cumulative effect of iron loss through menstrual blood can lead to iron deficiency over time. Addressing iron deficiency in individuals with heavy menstrual bleeding may require both iron-rich dietary strategies and, in some cases, medical interventions to reduce menstrual blood loss [31].

Physiological changes during pregnancy: Pregnancy induces significant physiological changes in the maternal body. One such change is an expansion of blood volume necessary to support the growing fetus. These increased blood volume requirements, coupled with the demands of fetal development, elevate the overall iron needs of pregnant individuals. As a result, the body may need to utilize iron stores to meet these increased demands. If maternal iron intake or absorption is insufficient to compensate for these changes, it can lead to the depletion of iron stores, potentially resulting in iron deficiency. This physiological adaptation underscores the importance of monitoring iron status during pregnancy and providing appropriate nutritional support to meet the increased iron requirements [32].

Influence of Comorbidities on Maternal Ferritin Levels

Chronic diseases: Several chronic diseases can disrupt the body's iron metabolism and lead to iron deficiency. Conditions such as inflammatory bowel disease (IBD), chronic kidney disease, and autoimmune disorders can affect the gastrointestinal tract, kidneys, and immune system, respectively, all of which are integral to iron regulation. In IBD, for example, intestinal inflammation can impair the absorption of dietary iron. At the same time, chronic kidney disease may reduce erythropoietin production, a hormone that stimulates red blood cell production. Autoimmune disorders can target iron-regulating proteins, disrupting the body's ability to maintain adequate iron levels. As a result, individuals with these chronic diseases may be at a higher risk of developing iron deficiency, necessitating careful monitoring and tailored interventions to address their specific iron needs [33].

Gestational conditions: Certain conditions, such as gestational diabetes and preeclampsia, can influence iron metabolism and ferritin levels during pregnancy. Gestational diabetes, characterized by insulin resistance and high blood sugar levels during pregnancy, may affect the regulation of iron metabolism. Preeclampsia, a condition marked by high blood pressure and organ dysfunction, can lead to oxidative stress and inflammation, potentially influencing the utilization of iron. Understanding the interplay between these gestational conditions and maternal iron status is essential for providing appropriate care and support to pregnant individuals. Regular monitoring of ferritin levels and tailored interventions may be required to address any disruptions in iron homeostasis associated with these conditions [34].

Infections and inflammation: Infections and chronic inflammation can significantly impact iron status through their influence on the hormone hepcidin. In response to inflammation or infection, the body often increases hepcidin production. This hormone inhibits iron absorption in the small intestine and reduces iron release from storage sites. This hepcidin-induced response can lead to functional iron deficiency, where the body's iron stores remain intact, but the iron cannot be adequately utilized. As a result, even individuals with sufficient iron stores can experience iron deficiency anemia. Recognizing the effects of infections and inflammation on iron regulation is vital for understanding the complexities of iron status and tailoring interventions to address inflammation-related iron disturbances [35].

Effects of Iron Supplementation on Ferritin Levels

Timing and dosage: The timing and dosage of iron supplementation are critical factors that can significantly influence ferritin levels in pregnant individuals. Adequate dosages of iron are necessary to replenish iron stores effectively. The timing of supplementation can also impact its absorption. For example, iron supplements are often recommended between meals or foods containing vitamin C to enhance absorption. Ensuring that iron supplements are taken as prescribed and optimizing their timing can maximize their effectiveness in raising ferritin levels and preventing or treating iron deficiency during pregnancy [36].

Adherence and tolerance: Compliance with iron supplementation is essential for its success. Pregnant individuals must adhere to the recommended dosages and duration of supplementation to achieve the desired improvement in ferritin levels. However, adherence can be challenging for some individuals due to side effects like nausea and gastrointestinal discomfort, which are common with iron supplements. These side effects can deter individuals from continuing to take their supplements regularly. Healthcare providers should be attentive to patients' tolerance and address any side effects by potentially adjusting the type or formulation of the iron supplement to improve adherence while maintaining adequate ferritin levels [37].

Monitoring and adjustments: Regular monitoring of ferritin levels is crucial during iron supplementation. This allows healthcare providers to track treatment progress and make necessary adjustments. Monitoring helps ensure that the intervention achieves the desired outcomes in ferritin levels to an appropriate range without causing iron overload, which can have adverse health consequences. Adjustments may include modifying the dosage of iron supplements or changing the type of iron supplement used based on the individual's response to treatment. Continuous evaluation and adaptation are key to effective iron supplementation strategies [38].

Clinical assessment of maternal ferritin levels

Methods for Measuring Maternal Ferritin Levels

Serum ferritin assay: Measuring serum ferritin levels in a blood sample is the most common and widely utilized method for assessing an individual's iron status. This assay provides a direct assessment of the concentration of ferritin in the bloodstream. Ferritin is primarily stored in the liver, spleen, and bone marrow, and a small amount circulates in the blood. By measuring serum ferritin, healthcare providers can estimate the total body iron stores, making it a valuable tool for diagnosing iron deficiency or overload. Serum ferritin assay results are typically expressed in nanograms per milliliter (ng/mL). They can guide clinical decision-making regarding the need for iron supplementation or further evaluation [39].

Enzyme-linked immunosorbent assay (ELISA): ELISA is a laboratory technique widely used for detecting and quantifying specific molecules, including ferritin, in serum or other biological samples. ELISA employs antibodies that selectively bind to ferritin, allowing for its detection. This method is highly sensitive and can provide precise measurements of ferritin levels. It is commonly used in clinical and research settings to assess iron status, monitor treatment responses, and investigate conditions related to iron metabolism [40].

Point-of-care testing: In clinical settings, point-of-care devices have been developed to enable rapid ferritin measurement. These tests provide quick results, often within minutes, which can be especially valuable for immediate decision-making in emergency or urgent care situations. Point-of-care ferritin tests are designed for ease of use by healthcare professionals and may require only a small blood sample. While they offer rapid results, they may have slightly reduced sensitivity compared to more complex laboratory assays, but they are highly useful for screening and preliminary assessments [41].

Liver biopsy: A liver biopsy may be performed in certain clinical situations, such as when there is a need to directly assess iron storage in the liver. A liver biopsy involves the removal of a small sample of liver tissue, which is then examined under a microscope. This invasive procedure can provide precise information about the amount of iron in the liver and is particularly valuable for diagnosing hereditary hemochromatosis, a condition characterized by excessive iron absorption and accumulation in the body. However, due to its invasiveness and associated risks, liver biopsy is typically reserved for specific cases when other non-invasive assessments, like serum ferritin measurements, are inconclusive or when a definitive diagnosis is essential [42].

Timing and Frequency of Ferritin Assessments During Pregnancy

First trimester: Initiating ferritin level assessments during the first trimester of pregnancy is valuable for establishing a baseline and gaining insights into the individual's iron status at the outset of gestation. This initial measurement can help identify preexisting iron deficiency or iron overload in pregnant individuals, enabling healthcare providers to tailor interventions accordingly. By detecting iron status early, healthcare professionals can implement dietary modifications or supplementation interventions to ensure optimal iron levels throughout pregnancy [43].

Regular monitoring: Routine monitoring of ferritin levels is a common practice during pregnancy, and the frequency of assessments is typically determined by clinical judgment. Pregnant individuals at higher risk of iron deficiency, including those with a history of anemia or known risk factors, may undergo more frequent ferritin assessments to track their iron status closely. Regular monitoring allows healthcare providers to observe trends in ferritin levels and make timely adjustments to interventions, ensuring that iron status remains within the recommended range [44].

Third trimester: Monitoring ferritin levels in the third trimester is of particular significance, as this period is associated with the highest iron demands during pregnancy. The growing fetus and the expansion of maternal blood volume place substantial pressure on maternal iron stores. Ensuring that ferritin levels are adequate in the third trimester is crucial to prevent iron deficiency anemia. This condition could have adverse consequences for both the pregnant individual and the developing fetus. Regular third-trimester assessments provide an opportunity to intervene promptly if ferritin levels are lower than desired [45].

High-risk situations: In high-risk pregnancies, comorbidities, or specific clinical concerns, more frequent ferritin assessments may be necessary to track iron status closely. High-risk pregnancies could involve factors such as a history of severe iron deficiency anemia, multiple pregnancies (e.g., twins or triplets), or underlying medical conditions that affect iron metabolism. In these situations, closer monitoring is essential to ensure that iron levels remain within safe and healthy ranges. It allows healthcare providers to make informed decisions regarding iron supplementation, dietary modifications, or other interventions tailored to the individual's unique needs and circumstances [46].

Interpretation of Ferritin Results in Clinical Practice

Reference ranges: Ferritin can vary depending on the laboratory and the specific population served. Healthcare providers should rely on the reference ranges provided by their respective laboratories, considering the demographics and characteristics of the population they are serving. Understanding the appropriate reference ranges is crucial for accurately interpreting ferritin levels and making informed clinical decisions. Deviations from the reference ranges may prompt further investigation into potential iron status abnormalities, guiding the implementation of appropriate interventions [47].

Low ferritin: Low ferritin levels often indicate iron deficiency, suggesting depleted iron stores in the body. However, the interpretation of low ferritin levels should be considered alongside other clinical parameters, such as hemoglobin levels and mean corpuscular volume (MCV), to comprehensively understand the individual's iron status. Incorporating additional hematological parameters can help differentiate between various types of anemia and contribute to a more precise diagnosis and tailored management approach [48].

High ferritin: Elevated ferritin levels can indicate either iron overload or inflammation. Distinguishing between these two possibilities is essential for appropriate management. While high ferritin levels can signal excess iron stores in the body, they can also be a marker of inflammation, as ferritin is an acute-phase reactant. Further evaluation, including additional laboratory tests and a thorough clinical assessment, is necessary to differentiate between iron overload and inflammation and to guide subsequent interventions effectively [49].

Clinical assessment: Interpreting ferritin results within the context of the individual's clinical status is crucial for a comprehensive understanding of their overall health. Healthcare providers should consider the results of other diagnostic tests, the individual's medical history, any relevant comorbidities, and concurrent conditions that may impact iron metabolism. Integrating these factors into interpreting ferritin results enables healthcare providers to make well-informed decisions regarding the appropriate course of action and develop personalized treatment plans [13].

Individualized care: The interpretation of ferritin results should serve as a guide for developing individualized care plans tailored to each patient's specific needs. Based on the interpretation of ferritin levels, healthcare providers can recommend dietary modifications, prescribe appropriate iron supplementation, or manage underlying health conditions contributing to abnormal ferritin levels. Personalized care plans not only address the immediate iron status concerns but also aim to optimize overall health outcomes and improve the individual's quality of life [50].

Interventions and strategies

Iron Supplementation During Pregnancy

Types of iron supplements: Healthcare providers have a range of iron supplements at their disposal, each with distinct characteristics. Commonly prescribed forms of iron supplements include ferrous sulfate, ferrous gluconate, and ferrous fumarate. The selection of the appropriate supplement depends on various factors, such as the individual's tolerance, absorption capabilities, and specific clinical considerations. For instance, some individuals may better tolerate one form of iron supplement over another due to differences in gastrointestinal side effects. Furthermore, the bioavailability of different iron compounds can vary, impacting their effectiveness in addressing iron deficiency. Healthcare providers must carefully consider these factors when determining the most suitable iron supplement for each patient [38].

Timing and dosage: The timing and dosage of iron supplementation should be tailored to the individual's iron status and clinical needs. Adequate dosages are necessary to address iron deficiency and replenish iron stores effectively. The timing of supplementation can also influence its absorption and tolerance. For example, iron supplements are often recommended between meals or foods containing vitamin C to enhance absorption. The choice of dosage and dosing schedule should align with the severity of iron deficiency, the individual's ability to tolerate the supplement, and other factors that may influence absorption, such as concurrent medication use. Individualized recommendations are vital to optimize the benefits of iron supplementation and minimize adverse effects [50].

Monitoring: Regular ferritin levels and hemoglobin monitoring are crucial components of iron supplementation. This ongoing assessment allows healthcare providers to track the individual's response to treatment and make necessary adjustments. Monitoring ferritin levels can help gauge the replenishment of iron stores while monitoring hemoglobin levels provides insights into improving anemia. These results guide clinical decision-making and may lead to dose adjustments, changes in the type of iron supplement, or alterations in the timing of supplementation. Monitoring ensures that iron status remains within the desired range and that the intervention effectively addresses iron deficiency. It also enables healthcare providers to address any adverse effects and make informed recommendations for the duration of iron supplementation [38].

Dietary and Lifestyle Recommendations to Improve Maternal Iron Status

Iron-rich foods: Encouraging the consumption of iron-rich foods is a fundamental dietary recommendation to help pregnant individuals meet their iron requirements. These foods include lean meats, poultry, fish, beans, lentils, tofu, and fortified cereals. Iron from animal sources (heme iron) is more readily absorbed by the body than iron from plant-based sources (non-heme iron). Educating pregnant individuals about heme and non-heme iron sources and the importance of including various foods in their diet can support healthy iron levels. Providing guidance on meal planning incorporating iron-rich options is essential to ensure adequate iron intake [51].

Absorption enhancers: Promoting the intake of foods rich in vitamin C, such as citrus fruits, strawberries, and bell peppers, can enhance iron absorption from plant-based sources. Vitamin C helps convert non-heme iron into a more readily absorbed form in the body. Conversely, pregnant individuals should be aware of iron inhibitors, like tea and calcium-rich foods, which can impede iron absorption. Advising on appropriate food combinations and meal timing can optimize iron absorption. For example, enjoying a vitamin C-rich fruit with a meal containing non-heme iron sources can improve iron utilization [52].

Balanced diet: Stressing the importance of a balanced diet is essential for overall maternal health and iron status. A well-rounded diet should include a variety of nutrients and food groups, ensuring that pregnant individuals receive adequate iron and essential vitamins, minerals, and macronutrients. A balanced diet promotes well-being and can help prevent or address iron deficiency. It is important to educate pregnant individuals about the significance of a diverse and nutritious diet that supports the growing needs of both the mother and the developing fetus [53].

Food safety: Educating pregnant individuals about safe food handling and preparation practices is crucial for reducing the risk of foodborne illnesses that can lead to iron deficiency. Foodborne illnesses can cause symptoms such as vomiting and diarrhea, which may result in increased iron loss from the body. Emphasizing proper food storage, cooking temperatures, and hygiene measures can help prevent these illnesses. Pregnant individuals should also be informed about foods to avoid during pregnancy, such as raw or undercooked seafood and unpasteurized dairy products, as these can carry a higher risk of foodborne pathogens [54].

Strategies for High-Risk Populations

High-risk assessment: Identifying pregnant individuals at high risk of iron deficiency is a critical step in providing targeted intervention to prevent preterm birth and its associated complications. High-risk populations may include individuals with chronic diseases like IBD, chronic kidney disease, or autoimmune disorders, as well as those with a history of previous preterm birth. These individuals are more likely to experience disruptions in iron metabolism and may require specialized care to address iron deficiency effectively. Healthcare providers should conduct comprehensive assessments, including medical history and diagnostic tests, to identify those at higher risk and initiate appropriate interventions [55].

Multidisciplinary care: Collaboration among healthcare providers from various specialties, such as obstetricians, hematologists, and nutritionists, is essential to deliver comprehensive care for high-risk populations. Multidisciplinary care ensures that pregnant individuals receive the most up-to-date and evidence-based interventions to address iron deficiency and mitigate the risk of preterm birth. Obstetricians can oversee prenatal care, monitor maternal and fetal health, and make informed decisions about interventions like iron supplementation. Hematologists can provide expertise in managing complex cases of iron deficiency and its underlying causes, while nutritionists can offer dietary guidance tailored to the individual's iron requirements. This collaborative approach maximizes the chances of successful intervention and positive outcomes for high-risk pregnant individuals [56].

Early detection and management: Early detection of iron deficiency and prompt intervention are key strategies to mitigate the risk of preterm birth and its complications in high-risk populations. Regular monitoring of iron status, including ferritin levels, in individuals at higher risk is crucial for early detection. Upon identifying iron deficiency, healthcare providers should initiate appropriate interventions, such as iron supplementation, dietary modifications, or addressing underlying medical conditions. Early and effective management can help replenish iron stores, improve overall health, and reduce the risk of preterm birth and its associated adverse outcomes in high-risk pregnant individuals [57].

Potential Future Interventions and Research Directions

Personalized medicine: The future of maternal care may involve personalized approaches to iron supplementation and dietary recommendations, considering genetic, clinical, and demographic factors. Advances in genetics and precision medicine can help identify individuals who are more susceptible to iron deficiency or at risk of iron overload. By tailoring interventions based on an individual's unique genetic profile and health characteristics, healthcare providers can optimize iron status and reduce the risk of preterm birth. Personalized medicine approaches have the potential to revolutionize maternal care, improving outcomes for both mothers and their infants [58].

Iron status biomarkers: Ongoing research and technological advancements may lead to the discovery and utilization of new biomarkers and techniques for assessing iron status during pregnancy. These innovations could provide more accurate and comprehensive evaluations of maternal iron stores, enabling healthcare providers to make more informed decisions about interventions. Developing novel biomarkers could improve the early detection of iron deficiency and facilitate timely management, reducing the risk of preterm birth and its associated complications [59].

Novel therapies: Research into innovative therapies, such as iron-chelating agents, holds promise for expanding the options available for managing maternal iron status during pregnancy. Iron-chelating agents can help manage iron overload, which can be a concern in certain medical conditions. As these therapies evolve and become more accessible, they may offer alternative approaches to maintaining optimal iron levels, particularly in high-risk populations [60].

Inflammation and infection: A deeper understanding of the complex interplay between iron status, inflammation, and infections during pregnancy will likely lead to targeted preventive measures. Research in this area may reveal new insights into the mechanisms by which inflammation and infections contribute to preterm birth and how iron status influences these processes. This knowledge can inform strategies to mitigate the impact of inflammation and infections on pregnancy outcomes, potentially reducing the incidence of preterm birth in at-risk populations [61].

Global health initiatives: Expanding research efforts and implementing public health initiatives in regions with high rates of preterm birth and maternal iron deficiency can significantly impact reducing preterm birth rates on a global scale. These initiatives may include raising awareness about the importance of iron status during pregnancy, providing access to iron supplements and fortified foods, and improving maternal healthcare. Collaborative efforts between governments, healthcare organizations, and international agencies can help address the root causes of preterm birth and iron deficiency in underserved populations [62].

Limitations and challenges

Methodological Limitations in Previous Studies

Retrospective designs: Many studies investigating the relationship between maternal ferritin levels and preterm birth have utilized retrospective designs, such as case-control studies or the analysis of existing medical records. While these designs can provide valuable insights, they have inherent limitations. Recall bias, for example, can affect the accuracy of self-reported data related to dietary habits, iron supplementation, and medical history. Additionally, historical data may not capture the dynamic changes in iron status during pregnancy. Ferritin levels can fluctuate throughout gestation, and a single measurement may not fully represent an individual's iron status at different stages of pregnancy. Retrospective designs should be interpreted cautiously, and prospective studies with real-time data collection can help overcome some limitations [63].

Selection bias: Selection bias can be a concern in studies exploring the association between maternal ferritin levels and preterm birth. This bias may occur when the study participants are not representative of the broader population of pregnant individuals. For instance, if a study primarily includes participants from a specific demographic, geographic region, or healthcare setting, the findings may not be generalizable to a more diverse population. Addressing selection bias often requires careful sampling techniques and the inclusion of a more diverse and representative study population to enhance the external validity of the results [64].

Confounding factors: Controlling for all potential confounding variables is challenging for research. Various factors, such as maternal comorbidities (e.g., diabetes, hypertension), socioeconomic status, and dietary habits, can influence both ferritin levels and the risk of preterm birth. Failing to adequately account for these confounding factors in the analysis can lead to spurious or misleading associations. Researchers must employ robust statistical techniques, such as multivariable analysis, to control for confounding variables as effectively as possible. However, it is important to acknowledge that some unmeasured or residual confounding may persist in observational studies [65].

Timing of ferritin assessment: The timing of ferritin assessments can vary across different studies, making it challenging to pinpoint the critical periods during pregnancy when iron status may impact the risk of preterm birth. Iron requirements and physiological changes during pregnancy evolve over time, with different stages having varying implications for maternal and fetal health. Therefore, understanding when ferritin levels were assessed during pregnancy is crucial for accurately interpreting the findings. Studies that assess ferritin at multiple time points and consider the dynamic nature of iron status during gestation may offer more comprehensive insights into the relationship between iron status and preterm birth [66].

Variability in Ferritin Levels and Preterm Delivery Risk

Individual variability: One of the key challenges in interpreting maternal ferritin levels is the substantial individual variability. Ferritin levels can differ significantly among individuals due to various factors, including genetics, dietary habits, and overall health. What may be considered a “normal” or “low” ferritin level can vary based on population characteristics and laboratory reference ranges. Therefore, it is important to consider the context of the population being studied and interpret ferritin levels within the appropriate reference range [67].

Gestational changes: Pregnancy induces significant physiological changes in the body, including alterations in iron metabolism. Ferritin levels can fluctuate throughout pregnancy, reflecting the dynamic nature of iron status. These changes can make it challenging to establish consistent benchmarks for ferritin levels during different stages of pregnancy. Researchers and healthcare providers must consider the gestational age at which ferritin measurements were taken to ensure accurate interpretation and clinical decision-making [68].

Multiple factors: Preterm delivery is a multifactorial outcome influenced by many variables, including maternal age, underlying health conditions, socioeconomic factors, and lifestyle choices. As indicated by ferritin levels, iron status represents just one facet of this complex web of contributing factors. Isolating the direct impact of ferritin on the risk of preterm birth can be challenging due to the interplay of these variables. Research studies must carefully control for these confounding factors to discern the specific role of ferritin in preterm birth risk [69].

Lack of causality: Many studies exploring the relationship between maternal ferritin levels and preterm birth demonstrate associations rather than establishing causality. Determining whether low maternal ferritin directly causes preterm birth is a complex endeavor, as observational studies cannot prove causation. Additional research is required to establish a causal link, including interventional studies and in-depth mechanistic investigations. These studies may help elucidate the pathways through which maternal iron status influences the risk of preterm birth [70].

Ethical Considerations in Conducting Research on Pregnant Women

Informed consent: Obtaining informed consent from pregnant research participants can be challenging due to the potential vulnerability of this population and the dynamic nature of pregnancy. Pregnant individuals may experience heightened emotions and physical discomfort, making it crucial for researchers to provide comprehensive information about the study's purpose, procedures, potential risks, and benefits. Researchers must ensure that informed consent is voluntary and that participants fully understand what their participation entails. It may also involve accommodating the changing circumstances of pregnancy, as participants' health and perspectives can evolve during the study [71].

Risk-benefit assessment: Researchers conducting studies involving pregnant individuals must carefully evaluate the risks and benefits associated with participation. This assessment is particularly important when interventions or changes in clinical practice are involved. The potential risks to both the pregnant participant and the developing fetus must be thoroughly considered and balanced against the potential benefits, such as contributing to maternal and fetal health advancements. Researchers should employ rigorous ethical review processes to ensure that the benefits of the research outweigh any potential harm [72].

Fetal health: Ensuring the safety and health of the fetus is paramount in pregnancy-related research. Research protocols should prioritize fetal well-being, and interventions or data collection procedures should be designed to minimize risks to the developing fetus. Regular monitoring and follow-up of fetal health should be included in the research plan to promptly address any concerns or adverse events. Fetal safety and well-being should be a fundamental ethical principle guiding research involving pregnant individuals [73].

Privacy and confidentiality: Protecting the privacy and confidentiality of pregnant research participants is essential, given the sensitive nature of pregnancy-related research. Researchers should implement robust data security and privacy measures to safeguard participants' personal and medical information. Additionally, data reporting and publication should be conducted in a way that prevents the identification of individual participants. Ensuring privacy and confidentiality promotes trust between researchers and participants, encouraging more pregnant individuals to engage in research activities [74].

Equity and inclusivity: Ethical research involving pregnant individuals should strive to include diverse populations to ensure that research findings apply to a broad range of pregnant individuals. Inclusivity is essential to avoid potential research findings that benefit or harm certain groups disproportionately. Researchers should actively engage with communities, stakeholders, and potential participants to foster diversity and ensure research is conducted with equity. This commitment to inclusivity aligns with the principles of justice and fairness in research ethics [75].

## Conclusions

In conclusion, this comprehensive review has shed light on the intricate relationship between maternal ferritin levels and the occurrence of preterm delivery during pregnancy. While it is evident that maternal iron status, as indicated by ferritin levels, plays a crucial role in maternal and fetal well-being, the link between low maternal ferritin and preterm birth remains a complex and multifaceted topic. We have identified key factors that influence maternal ferritin levels, including dietary intake, genetic and physiological variations, comorbidities, and iron supplementation. These findings have significant implications for clinical practice, suggesting the importance of assessing and monitoring ferritin levels in prenatal care, particularly for those at risk of iron deficiency. Public health initiatives should prioritize education and targeted interventions to improve iron status in high-risk populations. However, several unanswered questions persist, including the establishment of causality, the delineation of specific mechanisms, the search for more accurate biomarkers, and the development of innovative interventions. Future research in this area is crucial to enhance our understanding and further improve maternal and child health outcomes, particularly in the context of preterm birth.
